# Defining the *Schistosoma haematobium* kinome enables the prediction of essential kinases as anti-schistosome drug targets

**DOI:** 10.1038/srep17759

**Published:** 2015-12-04

**Authors:** Andreas J. Stroehlein, Neil D. Young, Aaron R. Jex, Paul W. Sternberg, Patrick Tan, Peter R. Boag, Andreas Hofmann, Robin B. Gasser

**Affiliations:** 1 Faculty of Veterinary and Agricultural Sciences, The University of Melbourne, Parkville, Victoria, Australia; 2 HHMI, Division of Biology, California Institute of Technology, Pasadena, California, USA; 3 Genome Institute of Singapore, Republic of Singapore; 4 Cancer and Stem Cell Biology, Duke-NUS Graduate Medical School, Republic of Singapore; 5 Faculty of Medicine, Nursing and Health Sciences, Monash University, Clayton, Victoria, Australia; 6 Structural Chemistry Program, Eskitis Institute, Griffith University, Brisbane, Australia

## Abstract

The blood fluke *Schistosoma haematobium* causes urogenital schistosomiasis, a neglected tropical disease (NTD) that affects more than 110 million people. Treating this disease by targeted or mass administration with a single chemical, praziquantel, carries the risk that drug resistance will develop in this pathogen. Therefore, there is an imperative to search for new drug targets in *S. haematobium* and other schistosomes. In this regard, protein kinases have potential, given their essential roles in biological processes and as targets for drugs already approved by the US Food and Drug Administration (FDA) for use in humans. In this context, we defined here the kinome of *S. haematobium* using a refined bioinformatic pipeline. We classified, curated and annotated predicted kinases, and assessed the developmental transcription profiles of kinase genes. Then, we prioritised a panel of kinases as potential drug targets and inferred chemicals that bind to them using an integrated bioinformatic pipeline. Most kinases of *S. haematobium* are very similar to those of its congener, *S. mansoni*, offering the prospect of designing chemicals that kill both species. Overall, this study provides a global insight into the kinome of *S. haematobium* and should assist the repurposing or discovery of drugs against schistosomiasis.

Schistosomiasis is a neglected tropical disease caused by blood flukes of the genus *Schistosoma* (phylum Platyhelminthes; class Trematoda)^1^,^2^. The three main species, *Schistosoma haematobium, S. mansoni* and *S. japonicum*, affect around 230 million people worldwide^1^. The former two flukes predominate, infecting almost 200 million humans in sub-Saharan Africa alone^3^,^4^. *S. haematobium* causes the urogenital form of this disease, and *S. mansoni* leads to hepato-intestinal illness^1^. These flukes have a complex life cycle, involving aquatic snails (family Planorbidae) as intermediate hosts. In freshwater, the infective larvae (cercariae) leave the snail and infect the definitive, human host by penetrating skin. Upon penetration, the cercariae lose their tails, and the larvae (schistosomules) migrate through the circulatory system and lung to the portal system, after which they mature and mate. Subsequently, paired adult worms migrate to their site of predilection and start to reproduce. *S. mansoni* adults live mainly in the portal system and/or the mesenteric venules of the small intestine, where they produce eggs that pass through the intestinal wall and are excreted in faeces. *S. haematobium* adults usually inhabit the blood vessels around the urinary bladder and genital system; here, the parasite produces eggs that pass through the bladder wall and are released in urine. Once eggs are released into freshwater, they immediately hatch to release miracidia (free-living larvae), which then invade a molluscan intermediate host^1^. *S. haematobium* infects snails of the genus *Bulinus*^5^, whereas *S. mansoni* prefers snails of the genus *Biomphalaria*^6^.

Disease in humans is precipitated by eggs that become entrapped in tissues, where they induce a chronic immune-mediated response, followed by granulomatous changes and ensuing fibrosis^1^. Eggs of *S. mansoni* become lodged mainly in the liver and intestinal wall, leading to egg-induced hepatitis, enteritis and/or associated complications^7^. In contrast, *S. haematobium* eggs are deposited mainly in the vasculature of the urinary bladder, ureter and/or genital tract (particularly in female individuals), although they can be disseminated to other sites in the body. Entrapped eggs induce considerable inflammation and subsequent fibrosis and/or calcification of the bladder. In addition, chronic *S. haematobium* infection can increase the risk of secondary bacterial infections^7^, is a predisposing factor for HIV/AIDS^8^ and can, together with other factors, induce malignant bladder cancer^9^. As there is no effective vaccine against schistosomiasis, current treatment relies on a single drug, praziquantel^10^. With increased efforts to control this disease by mass treatment, the possibility of praziquantel resistance developing is a serious concern^11^,^12^. Thus, there is a need for sustained research toward developing alternative chemotherapeutic compounds against schistosomiasis.

Recent research efforts to identify new molecular targets for chemotherapeutic intervention have focused on protein kinases^13^,^14^, because they are involved in signalling cascades of essential regulatory and developmental processes^15^,^16^,^17^, particular kinase groups have relatively conserved structures^18^, and also because drugs targeting these enzymes in humans have shown particular potential for the treatment of cancers and other diseases^19^,^20^. Protein kinases are enzymes (transferases) that phosphorylate a substrate by transferring a phosphoryl group from an energy-rich molecule, such as adenosine triphosphate (ATP), to a target protein. This phosphorylation induces a modification of the substrate, leading to changes in conformation and activity^21^. Substrates are phosphorylated at an amino acid residue that has a free hydroxyl group. Kinases can be subdivided into serine/threonine-phosphorylating kinases (STKs), tyrosine-phosphorylating kinases (TKs) and kinases that phosphorylate either of these residues (called ‘dual-specificity’ or ‘hybrid’ kinases). The conserved, catalytic domain of kinases is a protein fold consisting of an amino-terminal lobe comprised of β-strands and a carboxy-terminal lobe that contains α-helices^22^. A polypeptide linker functions as a hinge and connects the two lobes, allowing for rotation. This lobe structure forms a catalytic cleft for substrate and ATP binding^15^,^22^,^23^.

Eukaryotic protein kinases (ePKs) represent the largest class of enzymes that share the same protein kinase-like (PKL) fold^24^. Kinases that have catalytic activity but are not structurally similar to the PKL fold are classified as atypical kinases (aPKs)^15^. Protein kinases can be assigned to groups, families and subfamilies based on sequence similarity in their catalytic domains and the presence of accessory domains. The established classification scheme for kinases (http://kinase.com/kinbase)^16^ is based on that originally proposed by Hanks and Hunter^23^, and defines nine ePK groups.

Recognising their essential role in a range of regulatory processes and relatively conserved structure and function^25^,^26^,^27^, more than 20 ePKs have been investigated experimentally in *S. mansoni*^13^,^25^. Some of these kinases have been shown to assume essential functions in the parasite^17^,^28^,^29^,^30^,^31^. For example, the targeting of multiple receptor kinases of *S. mansoni* with a single inhibitor led to a fatal impact on schistosome morphology and physiology^32^. The fact that human protein kinases are involved in cancer and numerous compounds which inhibit these enzymes are available and approved for therapeutic use offers a unique prospect of repurposing such chemicals to schistosomes^25^. In this context, the *in silico* prediction of the kinome of *S. mansoni* provides a basis for the investigation of schistosome kinases as drug targets^33^.

In contrast to the situation for *S. mansoni*, there is no detailed information on the kinome of *S. haematobium* or any other schistosome. Given that *S. haematobium* is the causative agent of schistosomiasis in approximately two thirds of all humans infected by schistosomes and therefore has a substantial socioeconomic impact, in terms of disability-adjusted life years and morbidity^4^, there is a major need to work toward identifying drug targets in *S. haematobium* and designing new treatments^34^,^35^. In the present study, we defined the kinome of *S. haematobium*. Employing the *S. mansoni* kinome as a reference^33^, we: (i) curated the full complement of predicted kinases of *S. haematobium* using a comparative genomic-phylogenetic approach; (ii) assessed levels of transcription of genes encoding these kinases in the adult and egg stages of *S. haematobium*, and (iii) prioritised a panel of kinases as potential drug targets as well as chemicals inferred to bind to them using an integrated bioinformatic pipeline. We discuss the findings in the context of drug discovery and with regard to the distinctive biologies of *S. haematobium* and *S. mansoni*.

## Results

### The *S. haematobium* kinome

**Figure 1:**
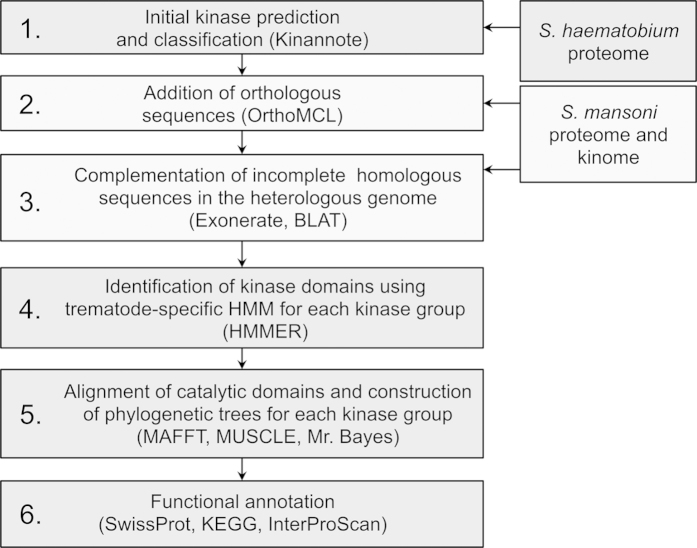
Bioinformatic pipeline used to characterize and curate kinases in *Schistosoma haematobium.* In step 1, we predicted and classified kinases in *S. haematobium.* In steps 2–3, additional sequences were identified employing the proteome^51^,^88^ and kinome^33^ inferred from the *S. mansoni* genome; incomplete or missing sequences were complemented using orthologous full-length sequences, which resulted in the final set of predicted kinase sequences. In steps 4 and 5, the catalytic domains in the kinase sequences were identified using trematode-specific HMMs for individual kinase groups, and then aligned (according to group) for subsequent phylogenetic analysis. In step 6, all kinases identified were functionally annotated employing SwissProt, KEGG and InterProScan databases.

**Figure 2:**
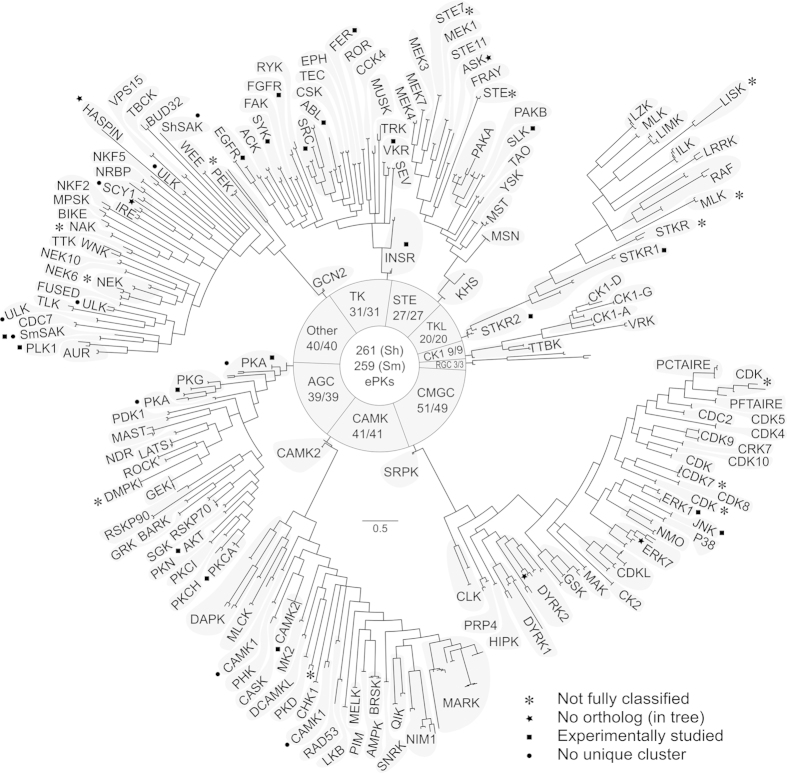
Phylogenetic analysis of eukaryotic protein kinases (ePKs) of *Schistosoma haematobium* and *S. mansoni*. Following the alignment of amino acid sequences representing individual kinase groups, phylogenetic trees were constructed. High resolution figures of individual trees including nodal support values and sequence identifiers are given in Supplementary Figs 2–9.

Here, we employed an integrative bioinformatic pipeline (Fig. 1). First, we predicted 223 kinases in the *S. haematobium* genome, 111 and 93 of which could be assigned to subfamilies and families, respectively; 10 could be assigned exclusively to a group, and nine remained unclassified. Subsequently, we identified 46 additional kinase sequences. Following this curation, the number of unclassified sequences decreased to four, and an improved classification of kinases into subfamilies (n = 134), families (n = 129) and groups (n = 2) was achieved (Fig. 2; Supplementary Table 1). Thus, the curated *S. haematobium* genome was inferred to encode 269 kinases, including both ePKs and PKLs.

A total of 261 ePKs representing all nine major kinase groups were identified in *S. haematobium* (Fig. 2; Supplementary Table 1). The largest group represented CMGCs (n = 51), including 17 cyclin-dependent kinases (CDKs), four CDK-like kinases (CDKLs), 10 mitogen-activated protein kinases (MAPKs), 11 dual-specificity tyrosine-regulated kinases (DYRKs), four glycogen synthase kinases (GSKs), two CDC-like kinases (CLKs), and one member of each of the families CK2, RCK and SRPK. The second largest group was CAMK, representing 41 kinases including CAMKs, CAMK-related kinases, MARKs and death-associated protein kinases (DAPKs). Only slightly smaller was the ‘Other’ group, which included 40 kinases representing 20 families that do not belong to any of the eight other ePK groups; this group included NEK, AUR (Aurora kinase), BUD32, HASPIN, two Polo-like kinases (PLKs), PEK (pancreatic eIF-2alpha kinase), SCY1 and ULK (Unc-51-like kinase). The AGC group represented 39 kinases, including the cyclic nucleotide-dependent kinase families PKA (n = 6) and PKG (n = 4), and PKCs (n = 5), RSKs (n = 5) and DMPKs (n = 7). Of the 31 members of the TK group, 13 were receptor tyrosine kinases (RTKs), including epidermal growth factor receptors (EGFRs), fibroblast growth factor receptors (FGFRs), insulin receptors (INSRs or IRs) and two venus kinase receptors (VKRs). The other 18 members were cytoplasmic tyrosine kinases (CTKs) and were assigned to 11 families (ABL, ACK, CSK, FAK, FER, RYK, SEV, SYK, TEC, TRK and SRC). The STE group contained 18 members of the STE20 family (MAP4Ks), two STE11 kinases (MAP3Ks) and six STE7 family members (MAP2Ks). The 20 representatives of the TKL group belonged to the families STKR (n = 7), MLK (n = 6), RAF (n = 3), LRRK (n = 1) and LISK (n = 3). We also identified nine kinases belonging to the CK1 group, including three members of the Tau tubulin kinase family (TTBK) and one vaccinia-related kinase (VRK). Finally, with only three members, the receptor guanylate cyclases (RGCs) represented the smallest group of ePKs in the *S. haematobium* kinome.

In addition to ePKs, we identified four PKLs: two *ri*ght *o*pen reading frame *k*inases, *Sh*-RIOK-1 (A_06019) and *Sh*-RIOK-2 (A_01816), and two representing the ABC1 family (A_02560 and A_01324). The *S. haematobium* genome also encodes four unclassified serine/threonine kinases, to which we assigned the following annotations based on similarity searches against the protein database Swiss-Prot: A_05753 - Cell cycle serine/threonine-protein kinase CDC5; A_08069 - Kinase suppressor of Ras 1 (KSR); C_01296 - Serine/threonine-protein kinase WNK1; Sh_Smp_017900.1 - Ribosomal protein S6 kinase (RSK).

All remaining kinase sequences (n = 265) were assigned to families and/or subfamilies, except for two sequences (A_03674 and A_04152) that could be classified only to a group level (i.e. CAMK and STE, respectively). In a phylogenetic analysis, sequence A_03674 clustered with A_07692 (predicted PKD kinase), albeit with a low nodal support (61%; Supplementary Fig. 2), and thus could not be assigned with confidence to any particular family. The homolog of sequence A_04152 (STE family member) in *S. mansoni* (Smp_146290.1) has been classified previously as a STE7 kinase^33^, but, according to the present analysis, it clustered with a kinase of the STE20 family and FRAY subfamily with 72% nodal support (Supplementary Fig. 6). Thus, sequence A_04152 was not classified to a family or subfamily level.

For 267 of the 269 kinases defined in *S. haematobium*, orthologs were identified in *S. mansoni* based on a comparative genomic approach and subsequent phylogenetic analyses. For two *S. haematobium* kinase sequences, no ortholog was found, in spite of exhaustive searching of the *S. mansoni* genome (A_01970; CMGC/MAPK/ERK7 and A_07508; CMGC/DYRK/DYRK2), suggesting their uniqueness to *S. haematobium*.

A comparison of the kinomes of *S. haematobium* and *S. mansoni* revealed a high overall sequence identity (82–92%), similarity (87–94%) and a relatively conserved length (0–7% difference) between pairs of kinases (Table 1). The degree of sequence similarity among individual kinase groups differed considerably, with kinases from the groups CK1 and RGC, and unclassified and PKL kinases, being, on average, more dissimilar compared with the other groups (Table 1). A pairwise sequence comparison of kinases of *S. haematobium* with human homologs revealed an average sequence similarity ranging from 60.9% (PKL) to 76.3% (CK1) for kinases that could be classified. For unclassified kinase sequences, we observed low sequence identity (35.1% on average) to their closest human homologs.

Subsequent phylogenetic analyses of ePKs of both *S. haematobium* and *S. mansoni* supported the orthology found between pairs of kinases of these two species. With the exception of the Polo-like kinase *Sh*-SAK, and representatives of the ULK, SCY1, PKA and CAMK1 families/subfamilies (Fig. 2; Supplementary Figs 2–4), orthologous sequences formed pairs in individual trees (Fig. 2; Supplementary Figs 1–11), consistent with their classification using an approach based on hidden Markov models (HMMs). Seven kinase sequences were excluded from phylogenetic analysis, because the catalytic domain of one or both representatives of the orthologous pair did not match the trematode-specific HMM. Six of these sequences were members of the family SCY1 (A_01858, Smp_176440.1 and Sh_Smp_156890.1) or HASPIN (Smp_Sh_A_07473, Sh_Smp_158950.1 and Smp_158950.1), which are part of the ‘Other’ kinase group. The seventh sequence (Smp_Sh_A_06810) was a member of the STE group, STE11 family and ASK subfamily.

**Figure 3:**
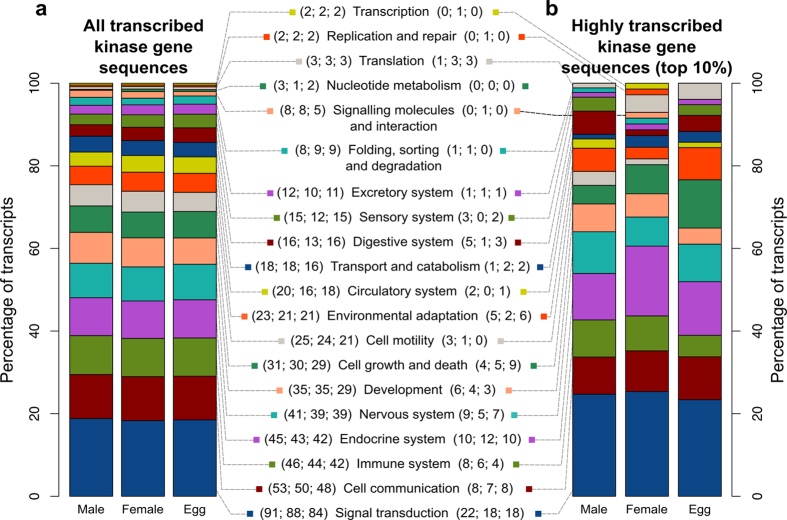
Functional annotation and levels of transcription of *Schistosoma haematobium* kinase genes. (**a**) All kinase genes transcribed in different sexes/developmental stages (male, female and egg). (**b**) Top 10% of transcribed kinase genes. Proteins inferred from these transcripts were associated with biochemical pathways. Numbers of inferred sequences in the respective functional category are indicated in parentheses for each sex/developmental stage.

Taken together, the 269 protein kinases of *S. haematobium* and 267 orthologs in *S. mansoni* were shown to represent all nine recognised kinase groups, 88 families and 79 subfamilies. However, we did not detect representatives of 19 kinase families and subfamilies (Table 2) in these two schistosomes (Lophotrochozoa; Protostomia) that are present in members of both the Ecdysozoa (Protostomia, represented by *Caenorhabditis elegans* and *Drosophila melanogaster*) and Deuterostomia (represented by *Homo sapiens*). Finally, we functionally annotated *S. haematobium* kinase sequences identified herein and linked them to 20 conserved functional categories (Fig. 3; Supplementary Table 1). Most kinases were predicted to have functional roles in signal transduction, cell communication, cell growth and the immune and/or nervous systems (Fig. 3; Supplementary Table 1).

### Transcription profiles

**Figure 4:**
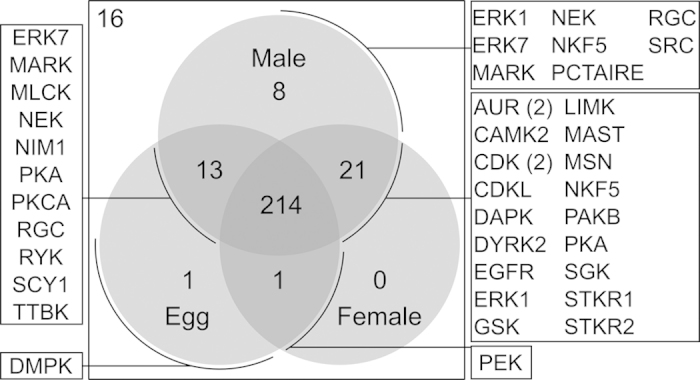
Venn diagram indicating the number of kinase genes selectively transcribed in the three developmental stages of *Schistosoma haematobium* studied. A total of 214 kinase genes were constitutively transcribed in all three developmental stages. Of the 274 coding regions, 16 were not transcribed. Kinase families/subfamilies assigned to transcribed kinase genes are indicated (boxed).

Following the curation and annotation of kinase sequences, we assessed transcription levels of respective genes in different developmental stages and genders of *S. haematobium* (adult male, adult female and egg). Of the 274 sequences encoding kinases identified in *S. haematobium*, 214 were transcribed in all three stages (Fig. 4). By contrast, 13 kinase genes were transcribed exclusively in the male and egg stages, 21 kinase genes were uniquely transcribed in the two adult stages, and one gene was transcribed in the female and egg stages, to the exclusion of the male stage (Fig. 4). One and eight kinase genes were transcribed exclusively in the egg and male stages, respectively. Among the eight male-specific genes were orthologs of the *t*estis-*ex*pressed gene *14* (*tex-14*, Sh_Smp_131630.1_p1) and a gene coding for an atrial natriuretic peptide receptor (A_02682), a kinase belonging to the RGC group that regulates cardiovascular and body fluid homeostasis^36^. For 16 kinase genes, there was no evidence of transcription in any of the life cycle stages studied here (Fig. 4; Supplementary Table 2).

We also assessed transcription levels for the four unclassified *S. haematobium* kinase genes. For the sequence A_05753, we did not observe transcription in any of the life stages studied; A_08069 was lowly transcribed in the adult female only (TPM: 0.06) and C_01296 was moderately transcribed in both adult stages (TPM female: 2.64; TPM male: 9.80); Sh_Smp_017900.1 was most highly transcribed in the egg stage (TPM: 50.97), but was also transcribed at varying levels in both adult stages (TPM female: 5.32; TPM male: 23.57).

Although most kinase genes were transcribed in all developmental stages of *S. haematobium* (Figs 3a and 4), there were differences in transcription levels, depending on their functional category (Fig. 3b). Notably, almost twice as many genes of kinases associated with cell growth and death were highly transcribed in the egg stage compared with either gender of the adult stage. In addition, kinase genes associated with cell motility were more abundantly transcribed in the male adult. We also found increased levels of transcription for kinase genes associated with environmental adaptation and the sensory system in the egg and male adult compared with the female adult stage.

### Druggable kinases and their prioritisation

**Figure 5:**
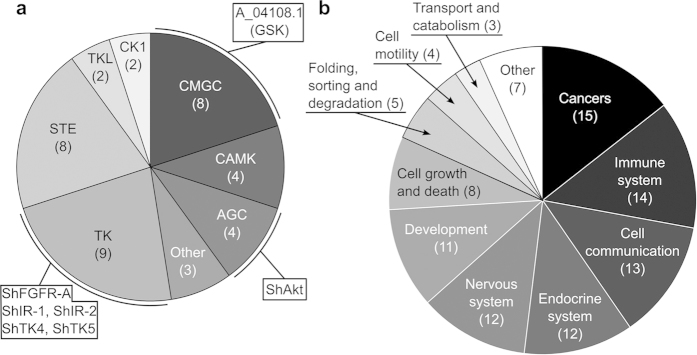
Kinases prioritised as targets in *Schistosoma haematobium* and associated pathways. (**a**) Numbers of predicted targets in individual kinase groups. Kinases that have already been investigated or prioritised in *S. mansoni* are indicated. (**b**) Pathway associations of prioritised targets.

Following the transcriptional analysis, we prioritised *S. haematobium* kinases as potential drug targets. First, we inferred the essentiality of *S. haematobium* kinase genes based on lethal gene knock-down or knock-out phenotypes linked to one-to-one orthologs in *C. elegans*, *D. melanogaster* and/or *Mus musculus* (Supplementary Table 3). In total, 219 of 269 (81%) *S. haematobium* kinases matched orthologs inferred to be associated with lethal phenotypes in at least one of the three organisms (Supplementary Table 3). Of these 219 kinases, 57 mapped (at amino acid level) to unique chokepoints in key biological pathways (Supplementary Table 3). Of these 57 kinases, 40 were predicted to bind chemical ligands listed in Kinase SARfari and DrugBank, 11 of which were present in both databases (Supplementary Tables 4–5). These 40 kinases represented all recognised groups, except RGC, and had human orthologs, some of which related to the nervous system, development and/or cancer (Fig. 5b).

Then, we showed that genes encoding these 40 kinases were transcribed in both adult and egg stages (n = 38), and that two (i.e. A_06570 and A_07448) were specific to adults (Supplementary Table 2). Amongst them were two casein kinases (A_08312.1 and Sh_Smp_099030.1) with >90% sequence similarity to human orthologs; four other kinases in this group (i.e. A_03569 (FAK), A_00551 (GCN2), m.56516 (RAF) and A_03539 (CHK1)) had ≤50% sequence similarity to human counterparts (Supplementary Table 1).

Of the 40 prioritised kinases, tyrosine kinases were the most highly represented group (n = 9), including a fibroblast growth factor receptor (*Sh*-FGFR-A), two insulin receptors (*Sh*-IR-1 and *Sh*-IR-2) and kinases SYK (*Sh*-TK4) and FYN (*Sh*-TK5), orthologs of which have been experimentally evaluated as drug targets in one or more schistosomes other than *S. haematobium*^13^,^29^,^32^,^37^,^38^,^39^. Two other targets, namely *Sh*-Akt (AGC group) and A_04108.1 (CMGC group; GSK family), were inferred, both of which have also been predicted to be promising drug targets in *S. mansoni*^40^,^41^ (Fig. 5a).

Taken together, we predicted that all 40 essential kinases represent targets, and therefore interrogated key databases for chemicals. We identified 42 drugs predicted to bind one or more of these targets, 17 of which are already approved by the FDA for the treatment of cancers or other diseases of humans (Table 3). These 17 drugs include four ABL kinase inhibitors (imatinib^42^, dasatinib^43^, bosutinib^44^ and ponatinib^45^), one JAK kinase inhibitor (tofacitinib), one GSK3 inhibitor (lithium carbonate), one protein kinase C inhibitor (ingenol mebutate) and 10 other drugs that inhibit multiple (receptor) kinases.

## Discussion

Here, we established an integrated bioinformatic pipeline to identify, classify and curate full-length kinase sequences encoded in the genome of *S. haematobium* for subsequent comparison with orthologs in *S. mansoni* and humans. This workflow enabled high-confidence predictions of anti-schistosome drug targets and compounds, and should be applicable to various schistosome species and, following modification, also to other flatworms as well as roundworms. In the future, we propose to gradually enhance the workflow by integrating tools for the prediction of binding sites of ligands, structural comparisons of prioritised targets and/or comparative analyses of parasite and host kinases into this pipeline.

In most previous studies, the identification of kinase sequences has relied on searches using HMMs from databases such as Pfam^46^ or Kinomer^47^, or position-specific scoring matrices (PSSMs)^48^. However, the combination of several of these methods can achieve enhanced predictions and classification compared with a single method. The program Kinannote uses such a combined approach, thereby increasing sensitivity and precision for kinase identification^49^, and was thus employed by us to produce a draft kinome in the first step of our workflow. Subsequently, an orthology-based approach^50^, using the published kinome^33^ and draft genome of *S. mansoni*^51^ as a reference, identified pairs of kinase orthologs, which facilitated the improvement of gene models for both schistosomes. This step also increased the number of kinases identified in *S. haematobium* by 17%, and their classification into families/subfamilies by 30%. Independent phylogenetic analyses verified the pairs of orthologs and functional subfamilies. Since the construction of reliable phylogenetic trees requires meticulous alignment of homologous characters, we restricted multiple alignments to the catalytic domains of kinases, because some sequence regions external to the catalytic domain can vary considerably. Phylogenetic trees calculated from these alignments can be used to sub-classify kinases, as sequence divergence in catalytic domains of kinases is recognised to reflect variation of function and/or mode of regulation of protein kinases^23^,^52^. The boundaries of kinase catalytic domains, such as *Pkinase* (Pfam identifier PF00069) or *Pkinase_Tyr* (Pfam identifier PF07714), are usually defined by HMMs. However, the sequences used to construct these two HMMs (n = 54 and n = 145, respectively) did not represent any lophotrochozoans, and thus, might not accurately represent the catalytic kinase domains of trematodes, which are clearly evolutionarily very distinct from those of Ecdysozoa and Deuterostomia^53^. In contrast to the alignment made using these Pfam HMMs, we obtained an improved alignment of homologous characters (with less gaps) by constructing a HMM from high-confidence kinase predictions for four trematode species.

Using the present bioinformatic workflow, we identified 269 full-length kinases that represent the kinome of *S. haematobium*. An assessment of transcription levels revealed transcription of 258 sequences, 214 (79.5%) of which were constitutively transcribed in all developmental stages/sexes studied, indicating essential roles for these kinases in signalling processes throughout the parasite’s life cycle. This statement is supported by the constitutive transcription of 83 of the 108 kinase genes (77%) assigned to the functional categories ‘signal transduction’ and/or ‘cell communication’. In contrast, only 11 (10%) kinase genes assigned to these general categories had variable transcription profiles. Although a small number of kinase sequences identified (n = 16; <6%) were not transcribed in either the egg or adult stage, they are likely to be transcribed in other developmental stages (including the miracidium, cercaria and/or schistosomulum) not investigated here. The validity of these sequences was supported by pairwise orthologs in *S. mansoni* that are transcribed in the cercarial and/or schistosomule stages^51^.

Sex-specifically transcribed kinase genes were more frequently assigned to specialised functional categories; among them was the male-specifically transcribed testis-expressed gene 14 (*Tex14*, Sh_Smp_131630.1), which we hypothesize is critical for chromosome segregation associated with mitosis and meiosis during spermatogenesis. This proposal is supported by findings in mice, showing that *Tex14* is highly expressed during spermatogenesis, and localises to intracellular bridges of germ cells, where it plays an integral role in the establishment and maintenance of male fertility^54^,^55^. Other evidence from a study of human cells lines shows that TEX14 is regulated by the kinase Plk-1 and is crucial for kinetochore-microtubule attachment during mitosis^56^.

A second gene encoding a protein kinase R (PKR)-like endoplasmic reticulum kinase (PERK; A_03220) was transcribed exclusively in female and egg stages of *S. haematobium*. The human ortholog of this kinase phosphorylates the eukaryotic translation initiation factor 2 alpha (eIF2α) and mediates the response to endoplasmic reticulum (ER) stress (represented by an accumulation of misfolded or unfolded proteins in the ER) which, among other factors, is induced by glucose deprivation^57^,^58^ and/or an excessive requirement for proteins^59^. The transcription of this additional, stress-mitigating kinase in eggs and female worms might thus be a mechanism to cope with increased ER stress due to the energy- and protein-demanding processes of reproduction, which are sustained by glucose metabolism. This specific transcription might also relate to stress on female worms, induced by separating them from their male partner (on which they rely, in terms of nutrient supply, such as sugar uptake from the host)^60^ prior to RNA-sequencing.

A third kinase gene encoding a myotonic dystrophy protein kinase (A_05067) of the DMPK family was transcribed exclusively in the egg stage of *S. haematobium*. Since different muscle types are already established in the miracidium within the egg, and a transformation of these muscle structures takes place during metamorphosis from sporocysts to cercariae^61^, we propose that this kinase-encoding gene is specifically transcribed in the miracidium in the egg, and is involved in muscle development and/or locomotion/motility. Evidence from other invertebrates, such as *D. melanogaster*, shows that DMPKs are involved in establishing correct muscle morphology and functionality in third instar larvae^62^. This aspect warrants further exploration when RNA-sequencing data for the miracidium stage of *S. haematobium* become available.

Comparative analysis showed that the *S. haematobium* kinome contains all recognised eukaryotic kinase groups, including 79 of the 144 (55%) subfamilies found in other metazoans studied^16^,^63^. The *S. haematobium* kinome has approximately half of the 518 kinases found in humans^15^ and has a similar number to that (n = 438) of the *C. elegans* kinome, to the exclusion of known specific expansions in this free-living nematode^16^,^63^. Nonetheless, we did not detect any members of 19 kinase families/subfamilies present in *C. elegans*, *D. melanogaster* or *H. sapiens*. The lack of evidence for kinases of these families/subfamilies, including RIO3 (which has been lost from numerous flatworms^64^), suggests their absence from schistosomes or a substantial diversification of their sequences that precluded their identification. Since there are presently no curated kinomes for flatworms other than *S. haematobium* and *S. mansoni*, it is not known whether such kinase families or subfamilies have been lost from all lophotrochozoans or only from schistosomes during evolution. A preliminary exploration of the flatworms *Clonorchis sinensis*, *Opisthorchis viverrini* and *Fasciola hepatica* (Stroehlein *et al.*, unpublished) suggests that these families and subfamilies (except the PIKK family) are absent from lophotrochozoans. Future studies should focus on defining and curating the kinomes of a range of socioeconomically important parasitic flatworms and roundworms (nematodes), in order to undertake detailed comparative analyses, explore kinome evolution and investigate contractions and expansions of particular kinase groups in relation to worm phylogeny as well as biology.

The global comparison of the kinomes of *S. haematobium* and its close relative, *S. mansoni*, did not detect any major expansions or contractions in kinase groups, families or subfamilies, but did reveal two kinase genes of the CMGC group (ERK7 and DYRK2 subfamilies) that are present exclusively in the former species. Given the quality of the draft genome and transcriptome of *S. mansoni*, there is only a remote possibility that these two genes were not detected. It is more plausible that they are indeed uniquely present in *S. haematobium* and encode kinases that may relate indirectly to this pathogen’s unique biology and site predilection in the human host. Published evidence indicating that ERKs are involved in parasite-host interactions^65^,^66^ supports this hypothesis. Although very little is known about the function of the second *S. haematobium*-specific kinase (DYRK2), in human and murine cell lines, a DYRK homolog interacts with the MAPK kinase MKK3 (an upstream activator of p38), which is involved in a growth factor-mediated signalling pathway^67^. The fact that both *S. haematobium*-specific kinases are part of receptor-activated signalling pathways advocates a role in pathogen-host interactions, as has been suggested previously for other receptor kinase pathways^68^,^69^.

Despite this difference of two kinases, the comparison of the kinomes of *S. haematobium* and *S. mansoni* showed a relatively high level of conservation of kinase sequences. Although such conservation has been reported previously for small numbers of kinases^32^,^38^,^70^, here we report the first global comparison of these kinomes. The conservation between the kinomes of the two most medically important species of schistosomes is considered to provide opportunities for the repurposing of existing, safe drugs against both species^25^. Thus, we focused on 40 *S. haematobium* kinase genes with (relatively) conserved orthologs in *S. mansoni* and *S. japonicum* (not shown) as well as human, whose gene products are inferred to be essential and to bind drugs available for treating human diseases.

A functional annotation of these 40 kinases showed that 37.5% (n = 15) were linked to human orthologs that are involved in cancer pathways, and a similar number of kinases (n = 14; 35%) were linked to roles in the immune system (Fig. 5b). Based on these findings, we suggest that associated anti-cancer/anti-inflammatory compounds should now be assessed as to their ability to disrupt normal schistosome growth, development and/or viability *in vitro*. In this context, a recent study has shown that blood components (such as serum albumin and α-1 acid glycoprotein) impede the deleterious effect of the drug imatinib on schistosomes *in vitro*, which should be considered in the experimental design of *in vitro* or *in vivo* experiments^71^.

A list of compounds (Table 3) revealed promising candidates for repurposing as schistosome kinase inhibitors. Many of these compounds have been predicted to target multiple kinases (targeted poly-pharmacology), a property that can increase the deleterious effect of a drug, thereby overcoming limited efficacy (due to redundancies in signalling pathways) associated with some single-targeted drugs^72^,^73^. Among the selected compounds were the anti-cancer drugs imatinib and dasatinib, the latter of which is assumed to target the Src/Fyn kinase SmTK5 in *S. mansoni*^13^. The orthologous kinase in *S. haematobium* (*Sh*-TK5) is one of the 40 prioritised targets in this study. Other selected targets of particular interest (Fig. 5a) include a Syk kinase (*Sh*-TK4), four receptor kinases (*Sh*-IR1, *Sh*-IR2, *Sh*-FGFR-A and B_00871), two members of the AGC group (*Sh*-Akt and A_01385) and a GSK3 kinase (A_04108.1). These kinases have either already been computationally predicted as drug targets in *S. mansoni*, or there is some experimental evidence indicating that orthologs in *S. mansoni* are essential and/or can be inhibited *in vitro*^13^,^27^,^29^,^32^,^37^,^38^,^40^,^41^,^65^,^68^, which lends additional support to our predictions. Furthermore, we predicted 32 additional kinases as potential targets for which no experimental information is yet available for schistosomes, including a TTK kinase (Sh_Smp_171610.1) and an eIF2α kinase ortholog (A_00551). Sh_Smp_171610.1 is an ortholog of a human kinetochore kinase, also known as Mps1 (Monopolar spindle 1), which plays an essential role in the spindle assembly checkpoint (SAC) pathway^74^. The prioritised eIF2α kinase ortholog is involved in mediating stress-response pathways, and several members of this kinase family are essential in *Plasmodium falciparum* (malaria parasite)^75^. Taken together, the high sequence similarity between schistosome kinases and the availability of kinase inhibitors for human orthologs offer great prospect with regard to the development of new anti-schistosome drugs.

In addition to the conserved kinase complement, there is also considerable merit in exploring selective kinase targets, namely those that are specific to schistosomes but absent from the mammalian host. For instance, the two genes encoding VKRs are specific to schistosomes and other Protostomia^32^,^76^, but absent from humans. Some functional studies of *S. mansoni* have shown that the compound tryphostin AG1024 kills schistosomula and adults *in vitro*^32^,^77^ by targeting schistosome VKRs and IRs. Given the sequence conservation of VKRs and IRs between *S. haematobium* and *S. mansoni* (97.3% and 93.8% similarity, respectively), this compound is likely to also kill the former species. In the context of identifying further schistosome-specific targets, four pairs of unclassified schistosome kinases identified here (Supplementary Table 1) were of interest, as they exhibited substantially lower sequence similarity to their human orthologs compared with *S. mansoni* orthologs. Three of these kinase-encoding genes were transcribed at varying levels in at least one of the sexes of the adult stage. We suggest that these results might assist in designing inhibitors for schistosomes, particularly if the premise is to target less conserved structural regions in a kinase outside of the conserved catalytic domain. This hypothesis warrants testing.

The curated set of kinases for *S. haematobium* as well as for its close relative, *S. mansoni*, might provide a stepping stone to fundamental studies of the biology of selected kinases in these worms. For instance, gene knockdown experiments by double-stranded RNA interference (RNAi)^78^ could be conducted on adult worms to validate the essentiality of subsets of kinases as drug targets in schistosomes. Combined with transcriptomic, proteomic and metabolomic investigations^79^,^80^,^81^ of treated *versus* untreated schistosomes, such studies could provide insights into the biological (e.g., signalling) pathways affected in the schistosome and also verify the specific knockdown of kinase genes and gene products. Moreover, in a similar manner, chemical knockdown experiments could confirm the specificity of the predicted and prioritised ligands *in vitro*^82^. Concordance between RNAi and chemical knockdown results would then provide confidence regarding the bioinformatic drug target/drug predictions made. Subsequently, compounds for which one or multiple targets have been validated and that have shown efficacy *in vitro* could then be investigated further in a hit-to-lead phase. At this point, chemical analogs could be produced to optimise target selectivity and minimize side effects on the host organism. Selected chemicals with specific binding to a kinase target but with limited selectivity (e.g., because of activity in mammalian host cells) might still serve as probes^14^ to explore kinase biology in the parasite.

In conclusion, we believe that the present bioinformatic investigation represents a step forward in the characterisation and curation of worm kinomes. The concordance in results between *S. mansoni* and *S. haematobium* (Fig. 2; Supplementary Table 1) as well as known lethal/adverse effects of some inhibitors against *S. mansoni* kinases^13^,^27^,^29^,^32^,^37^,^38^,^40^,^41^,^65^,^68^ suggest that some of our target and drug predictions are promising. However, we acknowledge that the prediction of drug targets and associated ligands represents a humble beginning to an often long and challenging route to validate new chemical entities (NCEs), to assess them in a preclinical context by *a*dministration, *d*istribution, *m*etabolism, *e*xcretion and *t*oxicity (ADMET) testing^83^,^84^,^85^, and, *via* clinical trials (phases I-III; http://www.phrma.org/innovation/clinical-trials)^86^, to develop one or more safe, effective and specific anti-schistosomal drugs. We hope that our bioinformatic pipeline will assist, at least in part, at the very beginning of this long and expensive discovery and development process.

## Methods

### Defining the *S. haematobium* kinome

We predicted, curated and annotated the protein kinase complement encoded in the published draft genome^87^ using an integrated bioinformatic pipeline in six steps (Fig. 1):

1. First, we identified ePKs and PKLs of *S. haematobium* using the program Kinannote^49^ employing the *-m* (metazoan) option. Predicted kinase sequences were then classified according to group, family and/or subfamily^16^,^63^. Sequences that could not be unequivocally classified using this approach were retained for subsequent curation.
2. Orthologous kinase sequences from both *S. haematobium* and *S. mansoni* were predicted by pairwise sequence comparison using the program OrthoMCL^50^, employing publicly accessible (SchistoDB v.3.0; http://schistodb.net/schisto/ and GeneDB v.5.2; http://www.genedb.org/) genomic and transcriptomic datasets^51^,^87^,^88^. Amino acid sequences that grouped with classified kinases, but were not predicted to be kinases using Kinannote, were added to a kinase group, family or subfamily based on their respective orthologous sequence (in the heterologous species) and included in subsequent analyses.
3. Then, we exhaustively searched all of the genomic and transcriptomic data available for *S. haematobium* and *S. mansoni*, to be able to complement any incomplete sequences and also to retrieve kinase-encoding sequences that had not been predicted previously for either or both schistosome species. If a full-length ortholog could not be inferred for the heterologous species, the kinase amino acid sequence was aligned to the genomic scaffold coding for the incomplete gene using the program BLAT^89^. This genomic region was then exhaustively searched for a full-length orthologous coding domain using the program Exonerate^90^ employing the multi-pass suboptimal alignment algorithm and the protein2genome:bestfit model. Refined gene predictions and protein translations were named according to their ortholog identifier (e.g., Sh_Smp_123456.1 and Smp_A_12345).
4. To increase the sensitivity of identification of kinase domains of schistosomes, we constructed HMMs for individual kinase groups based on the catalytic domains of high-confidence trematode kinase sequences (assigned to a subfamily by Kinannote) using the program HMMER v.3.1b1 (http://hmmer.janelia.org/). These HMMs were constructed using the inferred proteomic datasets of *S. japonicum*, *C. sinensis*, *O. viverrini* and *F. hepatica*^91^,^92^,^93^,^94^, and were then employed to query kinase sequences of individual groups of *S. haematobium* and *S. mansoni* and to identify catalytic kinase domains.
5. The catalytic domain sequences of all predicted kinases representing individual groups were aligned using the program MAFFT v.6.864b, employing the L-INS-i option^95^. Alignments were improved using the program MUSCLE v.3.7 (-refine option)^96^ and by subsequent manual adjustment, to optimise the alignment of homologous characters. The aligned sequences were then subjected to Bayesian inference (BI) analysis in the program MrBayes v.3.2.2 (ref. 97). Posterior probabilities (pp) were calculated, as recommended, using a mixture of models with fixed rate matrices, generating 1,000,000 trees and sampling every 100th tree. The initial 25% of trees were discarded as burn-in, and the others were used to construct a majority rule tree. Phylogenetic trees were drawn using the program FigTree v.1.4.1 (http://tree.bio.ed.ac.uk/software/figtree/).
6. Curated kinase sequences were functionally annotated by searching the databases Swiss-Prot (database release 01/2014)^98^ and KEGG BRITE (database release 03/2014)^99^ using BLASTP v.2.2.28+ (ref. 100) and an e-value cut-off of 10^−05^. Pfam domains and PANTHER families were predicted using the program InterProScan v.5–44.0 (ref. 101). In addition, sequence identities and similarities to *S. mansoni* and human kinase homologs (sequences accessed from KinBase, http://kinase.com/kinbase/FastaFiles/) were determined for *S. haematobium* kinases by pairwise comparison using the program EMBOSS Matcher v.6.3.1 (ref. 102).

### Transcription analysis

We assessed transcription in male and female adults as well as eggs of *S. haematobium* using publicly available RNA-seq data^87^. Data were filtered using the program Trimmomatic^103^ and aligned to the final sequences encoding kinases using Bowtie v.2.1.0 (ref. 104). Levels of transcription (numbers of transcripts per million, TPMs) were calculated using the software package RSEM v.1.2.11 (ref. 105). Kinase genes were considered as transcribed if at least 5 read pairs mapped to their coding regions and they had a TPM of >0. For each kinase gene, a relative measure of transcription was inferred by ranking individual genes from *S. haematobium* by their TPM values. The top and bottom 10% of transcribed genes were defined as being highly and lowly transcribed, respectively.

### Drug target prediction and prioritisation

To assess the druggability of individual predicted kinases and to prioritise them as potential targets in *S. haematobium*, essentiality was inferred by selecting *S. haematobium* proteins homologous (BLASTP; e-value ≤10^−5^) to *C. elegans*, *D. melanogaster* and/or *M. musculus* kinases with a lethal phenotype upon gene perturbation - listed in WormBase^106^, FlyBase^107^ and MGI^108^. Essential kinases were considered to represent metabolic chokepoints if only one gene was assigned to one KEGG orthologous gene (KO) term for a KEGG pathway. These kinases were then matched to homologous kinase sequences in the databases Kinase SARfari^109^ and DrugBank v.3.0 (ref. 110) using PSI-BLAST v.2.2.26+ employing an e-value cut-off of 10^−30^ (ref. 111). If both query and target sequence had the same kinase classification (using Kinannote), the sequence in the database had one or more ligands that met the Lipinski rule-of-five^112^ and was flagged as “medicinal chemistry friendly”, salient information on associated ligands (chemicals or small molecules) was extracted from the two databases and used to assess the druggability of the target. Prioritised kinases predicted to bind compounds approved by the FDA for use in humans or assessed in clinical trials, as indicated in Kinase SARfari (https://www.ebi.ac.uk/chembl/sarfari/kinasesarfari), were considered to have potential as drug targets. Kinases with entries in DrugBank were prioritised as drug targets if at least one associated small molecule (with a description of its properties) was found in this database.

## Additional Information

**How to cite this article**: Stroehlein, A. J. *et al.* Defining the *Schistosoma haematobium* kinome enables the prediction of essential kinases as anti-schistosome drug targets. *Sci. Rep.* **5**, 17759; doi: 10.1038/srep17759 (2015).

## Supplementary Material

Supplementary Figures 1-11

Supplementary Table 1

Supplementary Table 2

Supplementary Table 3

Supplementary Table 4

Supplementary Table 5

Supplementary Table 6

## Figures and Tables

**Table 1:** Pairwise comparisons of *Schistosoma haematobium* (*Sh*) kinase sequences with orthologs in *S. mansoni* (*Sm*) and human.

Kinase group^a^	Length ratio *Sh*/*Sm* [SD]	*S. mansoni*% identity [SD]	*S. mansoni*% similarity [SD]	*H. sapiens*% identity [SD]	*H. sapiens*% similarity [SD]
CMGC	1.00 [0.08]	91.87 [6.10]	94.42 [4.96]	61.04 [8.93]	75.91 [7.15]
CAMK	1.00 [0.08]	87.89 [8.48]	91.48 [6.55]	54.57 [13.91]	71.31 [10.79]
AGC	0.99 [0.19]	91.20 [5.91]	93.78 [4.94]	52.40 [13.22]	68.66 [11.15]
Other	0.98 [0.17]	87.18 [8.46]	90.28 [7.77]	43.54 [12.06]	62.11 [10.87]
TK	0.99 [0.11]	87.66 [8.18]	91.12 [6.92]	45.74 [6.50]	63.68 [6.46]
STE	1.04 [0.22]	90.75 [6.89]	93.21 [6.22]	56.94 [12.14]	72.62 [10.73]
TKL	1.01 [0.08]	88.59 [6.33]	92.01 [5.67]	45.29 [9.95]	63.58 [8.68]
CK1	0.98 [0.14]	85.05 [8.21]	87.85 [7.94]	62.61 [11.20]	76.30 [8.58]
RGC	1.02 [0.19]	85.30 [6.00]	87.53 [6.86]	50.43 [5.36]	66.70 [5.38]
PKL	1.07 [0.12]	88.45 [3.92]	91.53 [4.01]	43.55 [6.34]	60.90 [5.82]
Unclassified	1.06 [0.11]	82.50 [14.84]	88.92 [9.30]	35.12 [7.76]	58.10 [3.93]

Average length ratios, identity and similarity values are indicated. Amino acid sequence conservation between *S. haematobium* and *S. mansoni* was observed for all kinase groups. Predicted sequences had very similar lengths. The comparison with human homologs showed moderate to low identities and similarities. Average and standard deviation [SD] values were calculated based on the number of predicted *S. haematobium* sequences in each kinase group.

^a^CMGC = Cyclin-dependent kinases (CDKs), mitogen-activated protein kinases (MAP kinases), glycogen synthase kinases (GSKs) and CDK-like kinases; CAMK = Ca^2+^/calmodulin-dependent kinases; AGC = Nucleoside-regulated kinases; TK = Tyrosine kinases; STE = MAPK cascade kinases; TKL = Tyrosine kinase-like kinases; CK1 = Casein kinase 1 kinases; RGC = receptor guanylate cyclases; PKL = Protein kinase-like kinases.

**Table 2:** Kinase families and subfamilies absent from the kinomes of *Schistosoma haematobium* and *S. mansoni* (Lophotrophozoa; Protostomia).

Name	Kinase classification
Novel (Nua) kinase family	CAMK/CAMKL/NUAK
MAPK-integrating or -interacting kinase	CAMK/MAPKAPK/MNK
Testis-specific serine/threonine kinase	CAMK/TSSK
RSK-like kinase	AGC/RSKL
Mitogen- and stress-activated protein kinase	AGC/RSK/MSK
RSK-related kinase	AGC/RSKR
Yet another novel kinase	AGC/YANK
Budding uninhibited by benzimidazoles kinase	OTHER/BUB
New kinase family 1	OTHER/NKF1
Anaplastic lymphoma kinase	TK/ALK
Discoidin domain receptor kinase	TK/DDR
IL1 receptor-associated kinase	TKL/IRAK
Serine/threonine-like kinase	STE/STE20/STLK
Eukaryotic elongation factor 2 kinase	ATYPICAL/ALPHA/EEF2K
Bromodomain-containing kinases	ATYPICAL/BRD
Pyruvate dehydrogenase kinase	ATYPICAL/PDHK
Phosphatidylinositol 3 kinase-related kinase	ATYPICAL/PIKK
Right open reading frame kinase 3	ATYPICAL/RIO/RIO3
TATA-binding protein-associated factor 1	ATYPICAL/TAF1

Members of these families and subfamilies are found in both Ecdysozoa (Protostomia) and Deuterostomia.

**Table 3:** List of prioritised chemical compounds as drug candidates against *Schistosoma haematobium*.

Name or code of compound	Number of target kinases	Indicated for treatment of	Status of approval
AG-13736 (axitinib)	1	Cancer	A
Dasatinib	32	Cancer	A
Pazopanib	30	Cancer	A
Erlotinib	30	Cancer	A
Imatinib	30	Cancer	A
Gefitinib	30	Cancer	A
Sorafenib	32	Cancer	A
Sunitinib	32	Cancer	A
Vandetanib	30	Cancer	A
CP-690550 (tofacitinib)	30	Rheumatoid arthritis, psoriasis, inflammatory bowel disease	A
Bosutinib	4	Cancer	A
Cabozantinib	1	Cancer	A
Ingenol mebutate	1	Cancer, actinic keratosis	A
Ponatinib	3	Cancer	A
Regorafenib	2	Cancer	A
Trametinib	2	Cancer	A
Lithium carbonate	1	Bipolar disorder	A
ABT-869 (linifanib)	31	Cancer	III
Vatalanib	30	Cancer	III
AMG-706 (motesanib)	30	Cancer	III
PD-184352	4	Cancer	II
PHA-739358 (danusertib)	6	Cancer	II
Seliciclib	30	Cancer	II
SNS-032	30	Cancer	I
Fasudil	4	Cerebral vasospasm, pulmonary hypertension	II
Ruboxistaurin	33	Diabetic retinopathy	III
CHEMBL1173486	2	Unknown	N/A
CHEMBL1230122	1	Unknown	N/A
CHEMBL150504	1	Unknown	N/A
AT7519	1	Cancer	II
AZD2171 (cediranib)	1	Cancer	III
CYC116	1	Cancer	I
Ellagic acid	2	Cancer	N/A
XL228	1	Cancer	I
XL518 (cobimetinib)	2	Cancer	III
XL820	1	Cancer	II
XL844	2	Cancer	I
XL880 (foretinib)	1	Cancer	II
XL999	2	Cancer	II
CEP-1347	1	Asthma, Parkinson’s disease	III
KC706	1	Rheumatoid arthritis, psoriasis, inflammatory bowel disease	II
TG100801	1	Macular degeneration, diabetic retinopathy	II

For each compound, the number of target kinases, its indicated therapeutic use(s) and the status of FDA approval for use in humans are given (A = approved; I, II or III = phase of clinical trial). Additional information and chemical structures are given in Supplementary Tables 4–6.
