# Supplementary material for: Defining the Schistosoma haematobium kinome enables the prediction of essential kinases as anti-schistosome drug targets
Source: Sci Rep. 2015 Dec 4;5:17759. doi: 10.1038/srep17759 (PMC4669435; doi:10.1038/srep17759)

Supplementary Figures

Defining the *Schistosoma haematobium* kinome enables the prediction of essential kinases as anti-schistosome drug targets

Andreas J. Stroehlein[1], Neil D. Young[1], Aaron R. Jex[1], Paul W. Sternberg[2], Patrick Tan[3,4], Peter R. Boag[5], Andreas Hofmann[1,6] & Robin B. Gasser[1]

[1]Faculty of Veterinary and Agricultural Sciences, The University of Melbourne, Parkville, Victoria, Australia, [2]HHMI, Division of Biology, California Institute of Technology, Pasadena, California, USA, [3]Genome Institute of Singapore, Republic of Singapore, [4]Cancer and Stem Cell Biology, Duke-NUS Graduate Medical School, Republic of Singapore, [5]Faculty of Medicine, Nursing and Health Sciences, Monash University, Clayton, Victoria, Australia, [6]Structural Chemistry Program, Eskitis Institute, Griffith University, Brisbane, Australia

Supplementary Figures 1 to 11. Phylogenetic analysis of individual eukaryotic protein kinase (ePK) groups and protein kinase-like (PKL) families of *Schistosoma haematobium* and *S. mansoni*. Following the alignment of amino acid sequences representing CMGC, CAMK, AGC, Other, TK, STE, TKL, CK1, RGC groups and RIO and ABC kinase families, phylogenetic trees were constructed. High-resolution figures of individual trees including nodal support values and sequence identifiers are given.

Supplementary Figure 1. CMGC kinase group

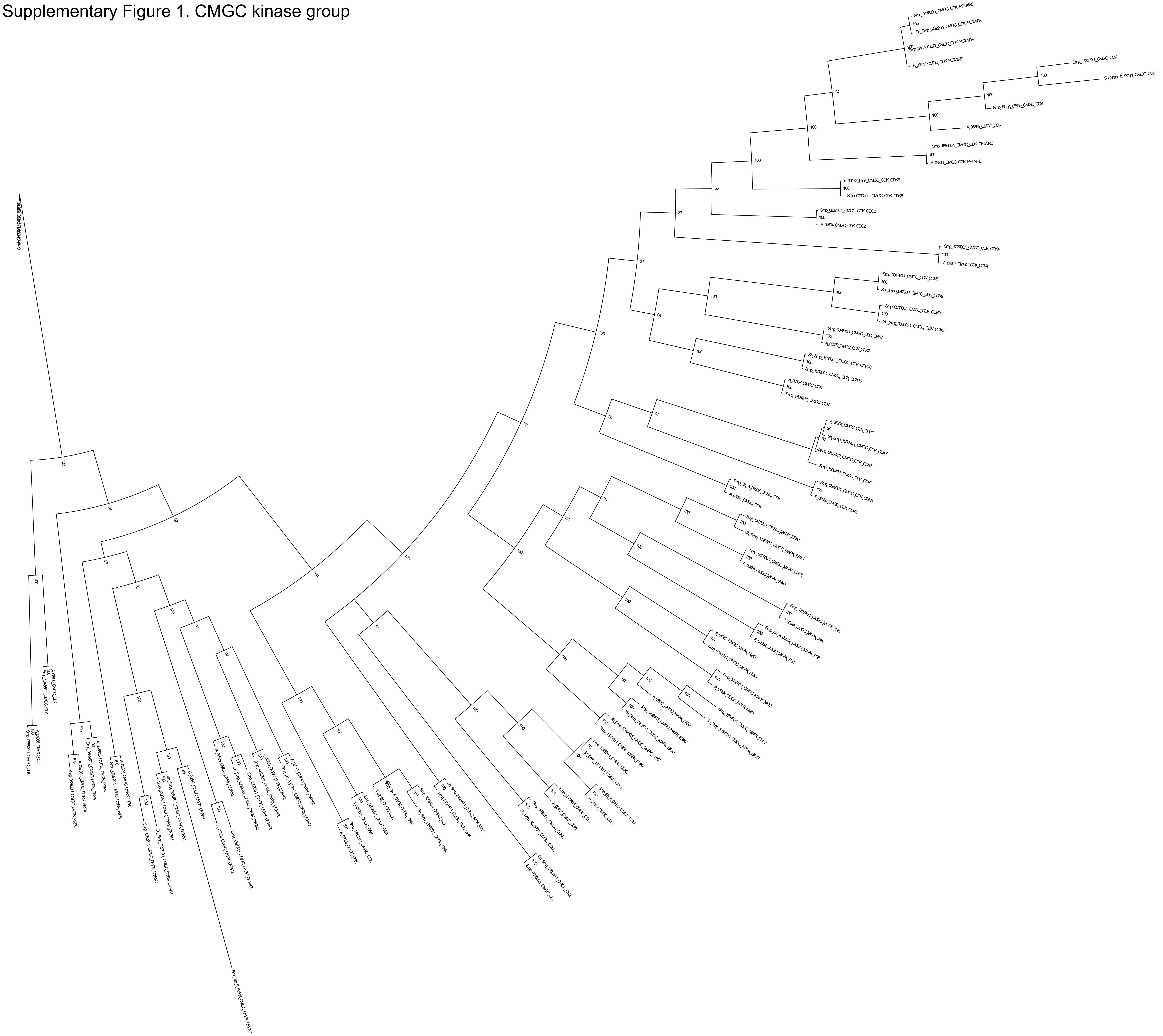

Supplementary Figure 2. CAMK kinase group

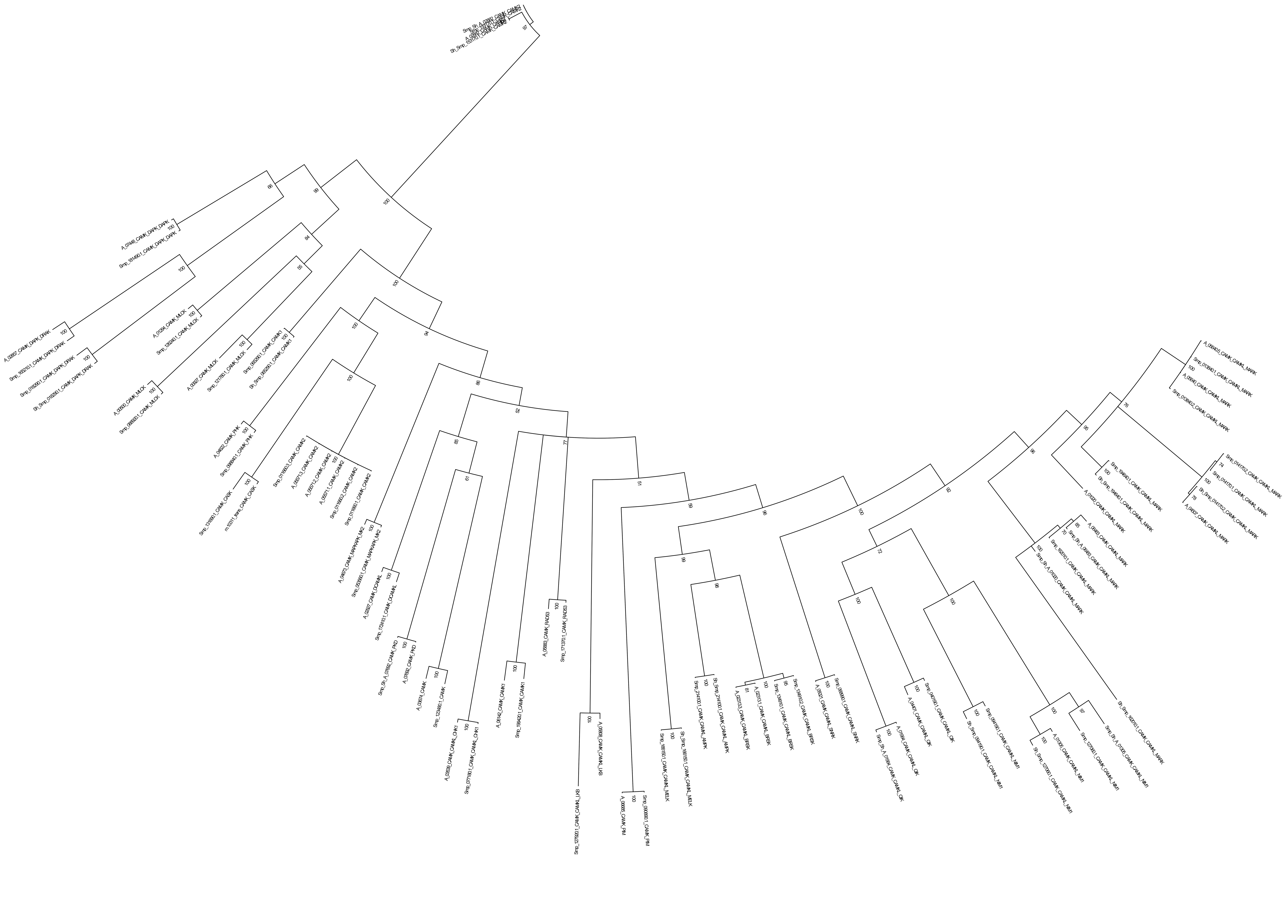

Supplementary Figure 3. AGC kinase group

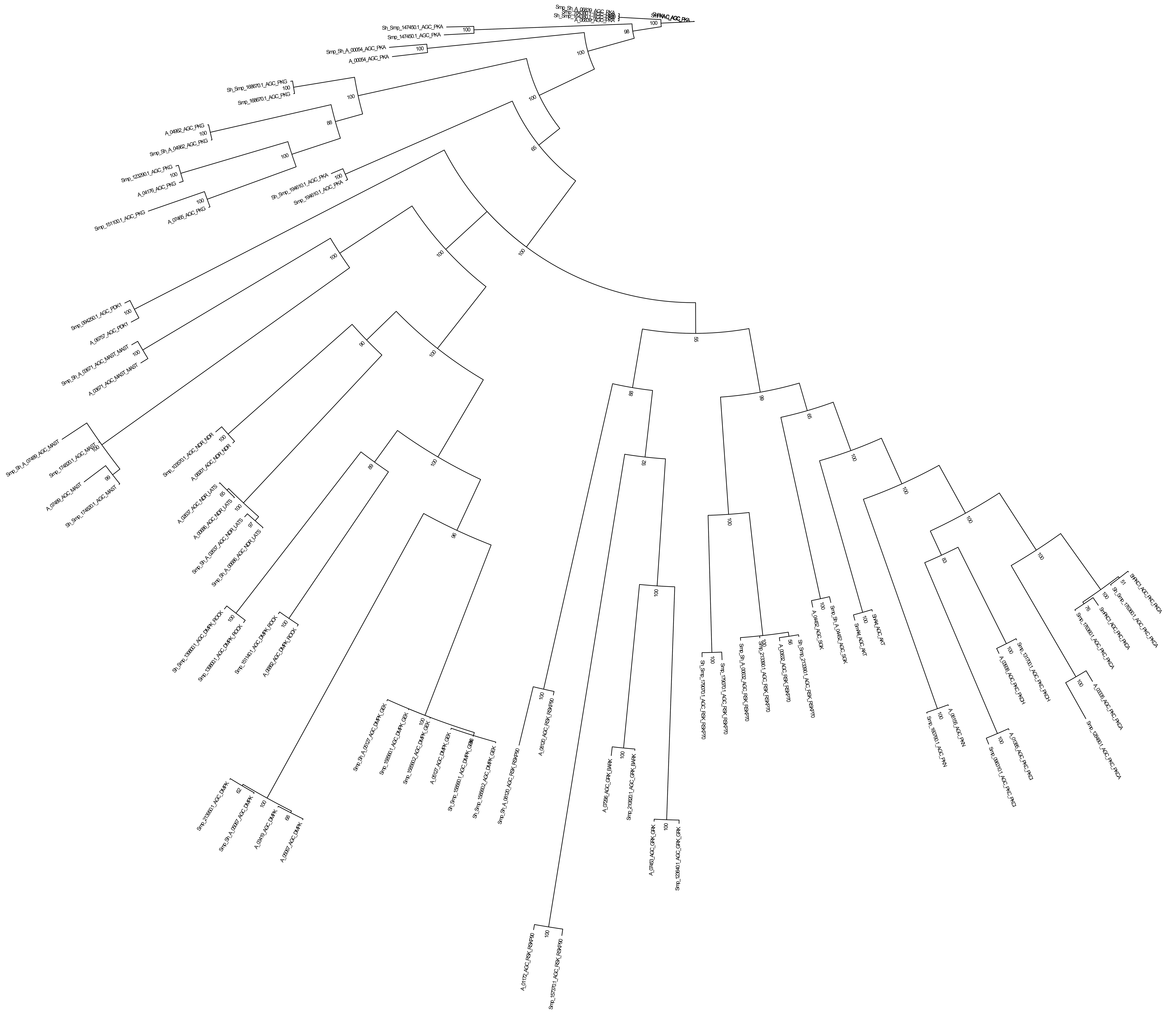

Supplementary Figure 4. Other kinase group

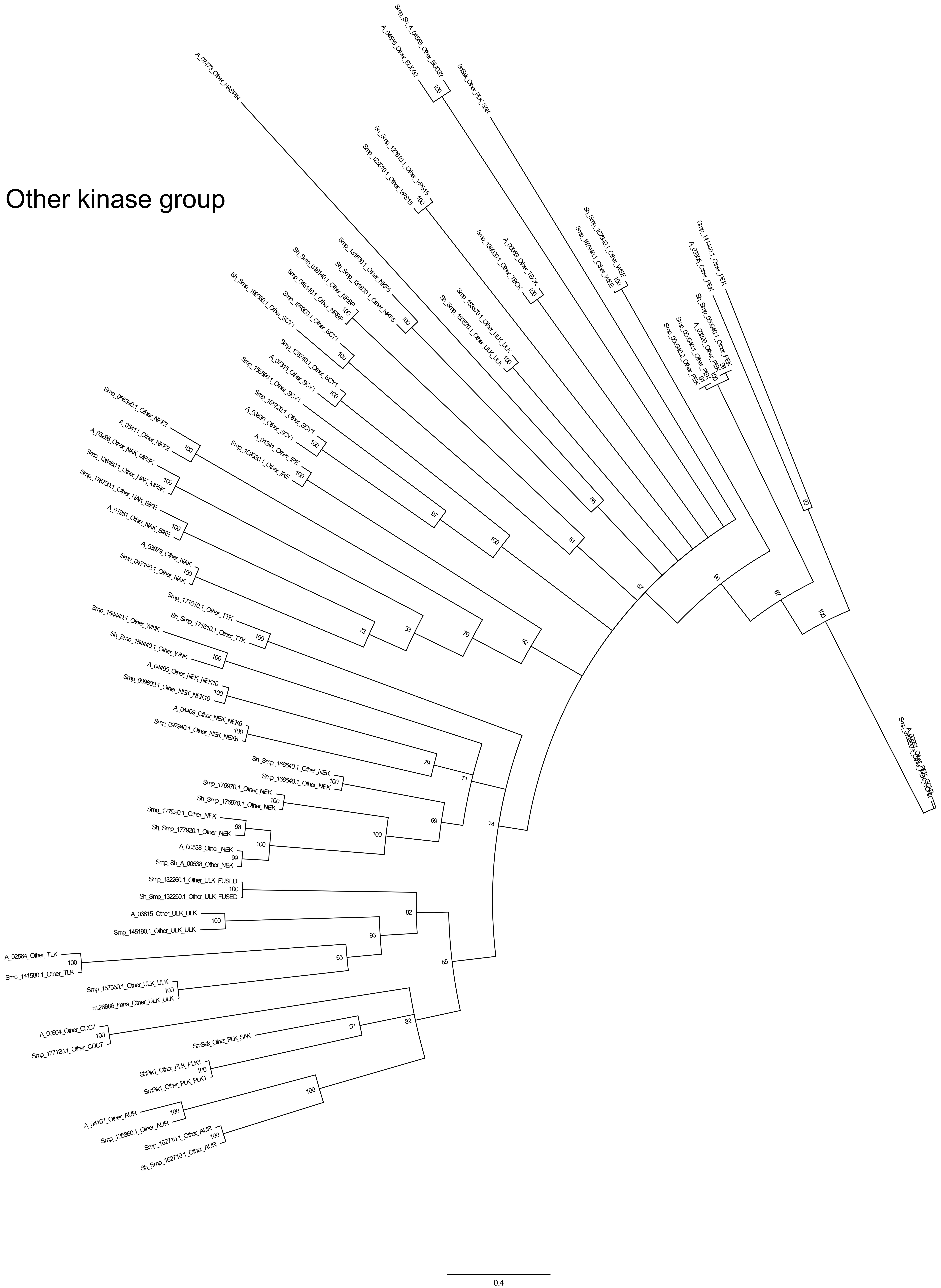

Supplementary Figure 5. TK kinase group

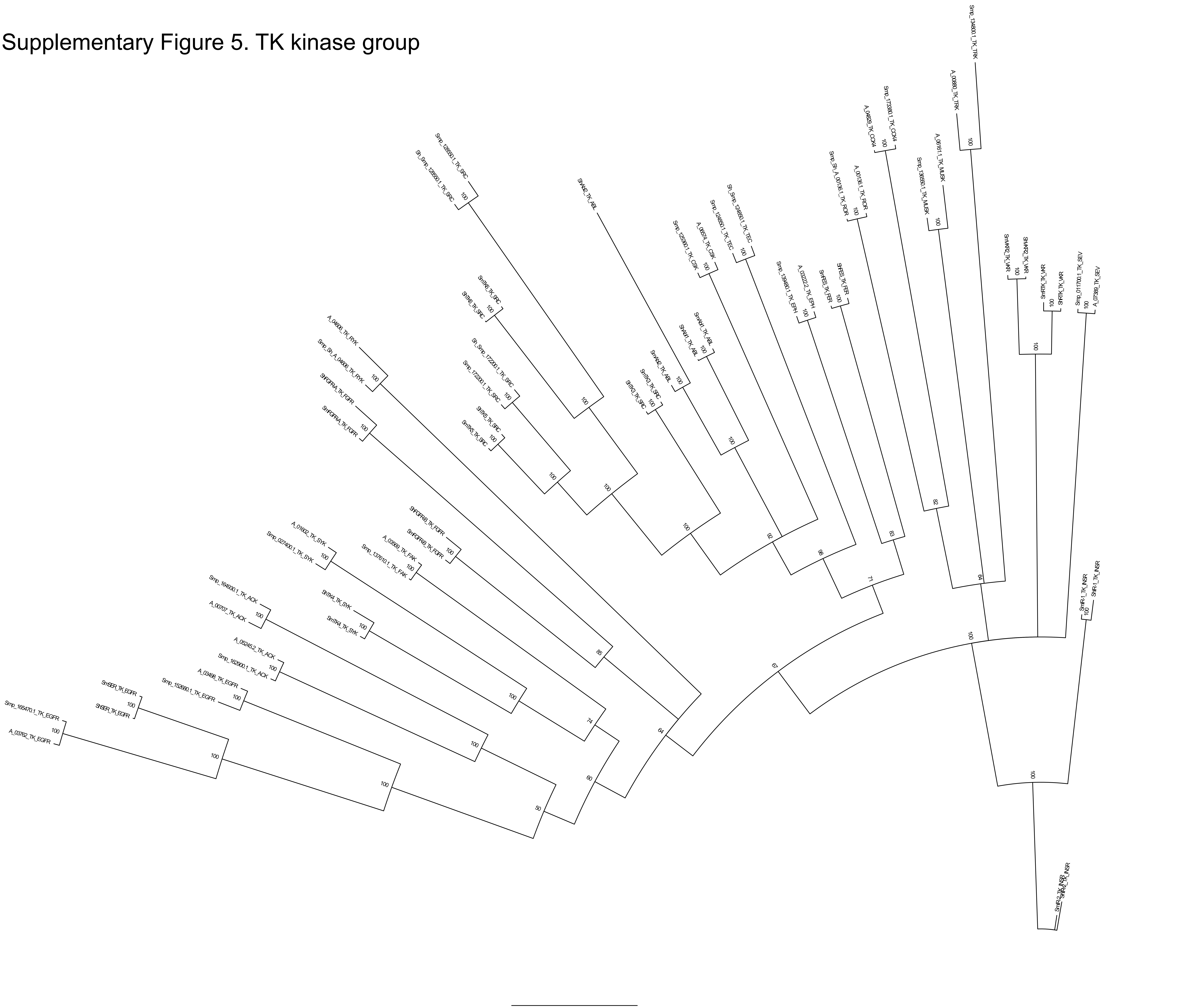

Supplementary Figure 6. STE kinase group

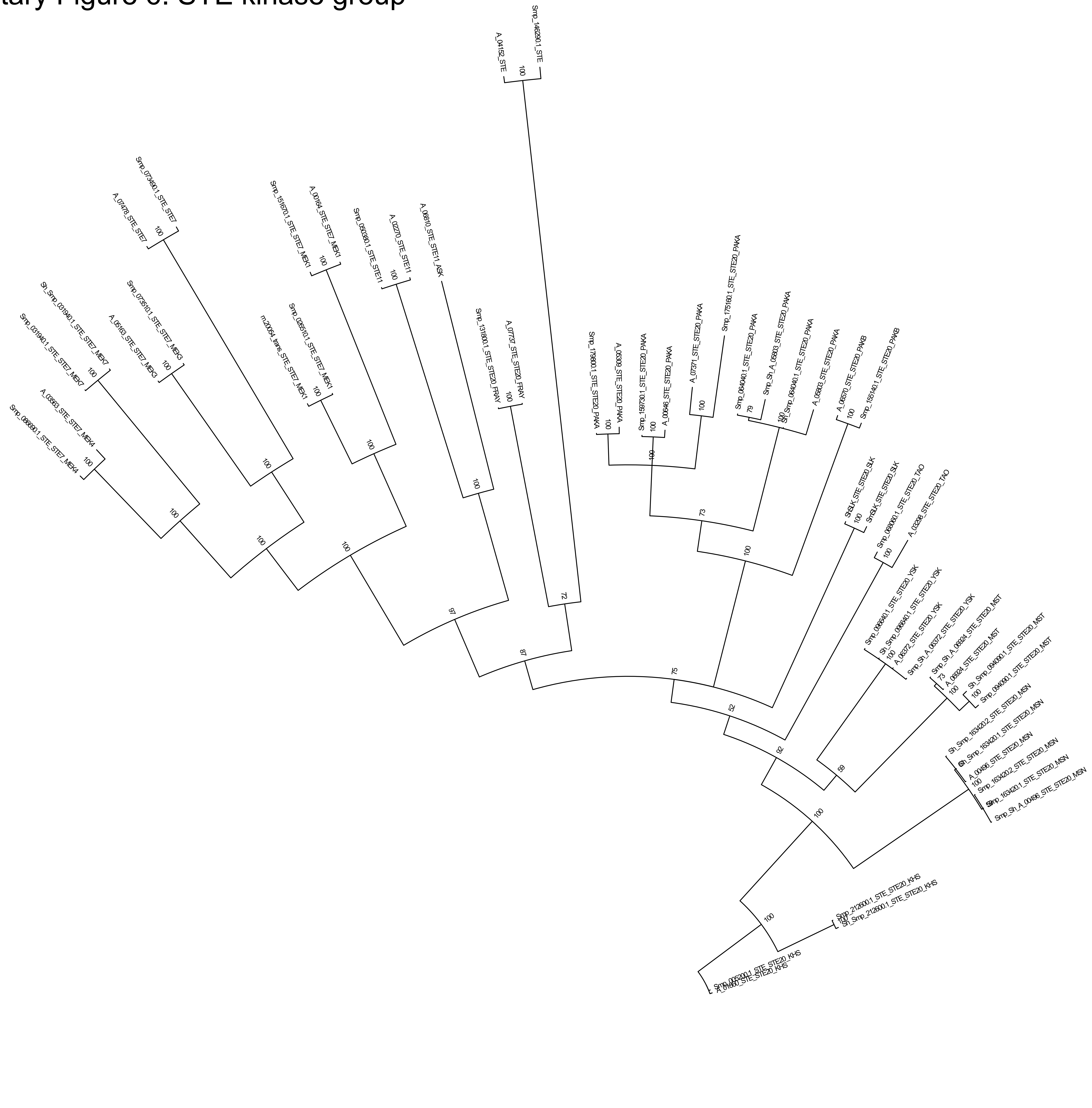

Supplementary Figure 7. TKL kinase group

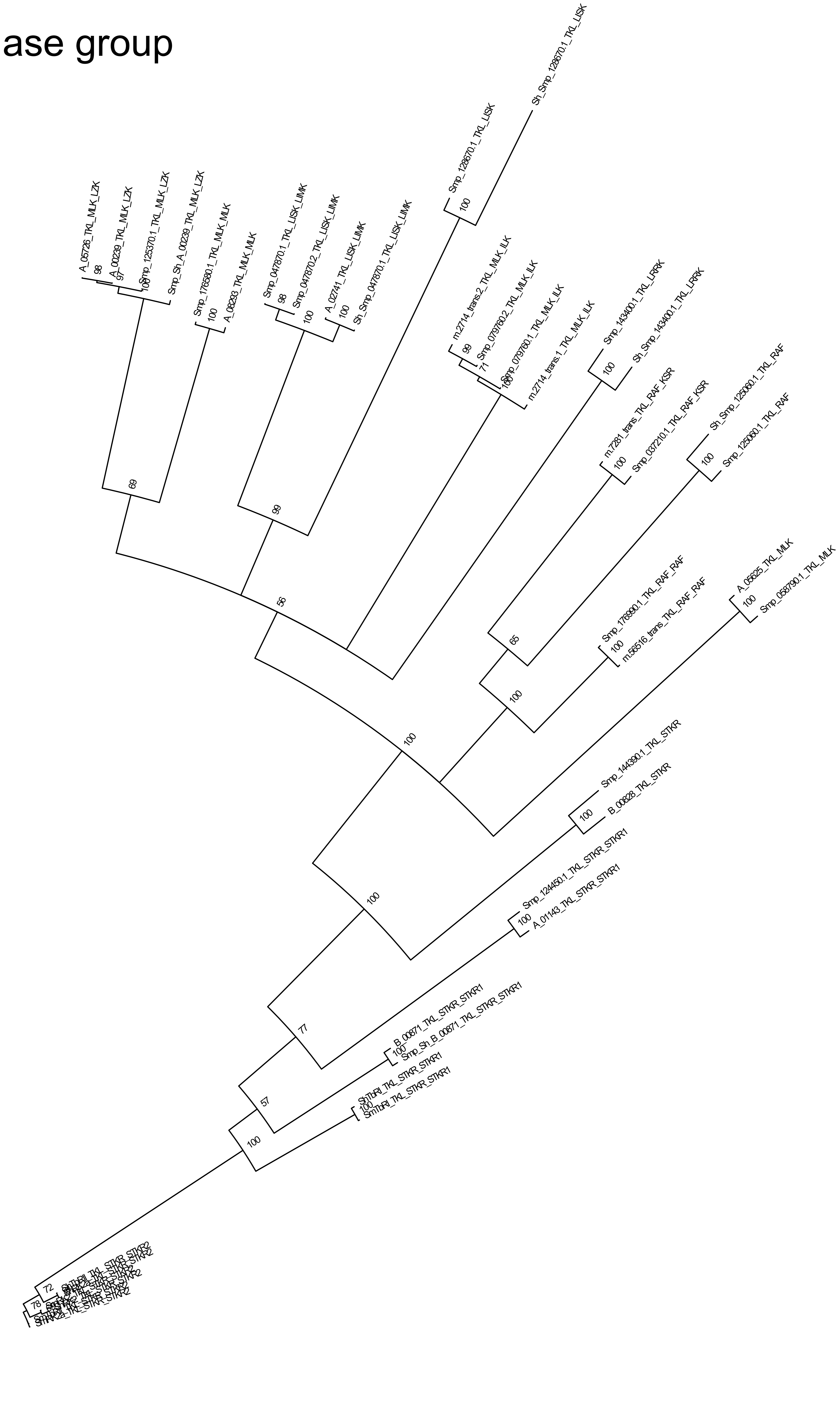

Supplementary Figure 8. CK1 kinase group

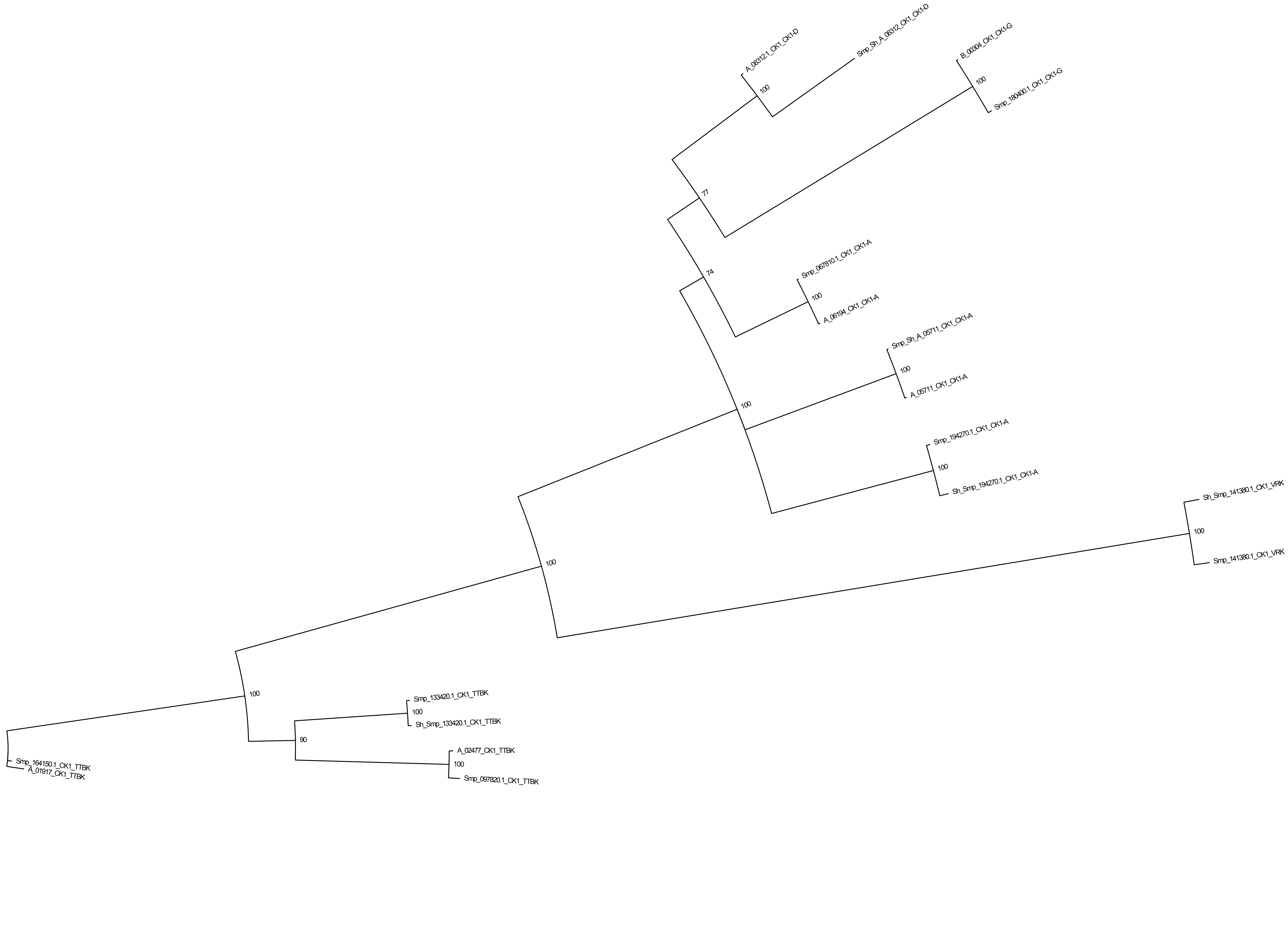

Supplementary Figure 9. RGC kinase group

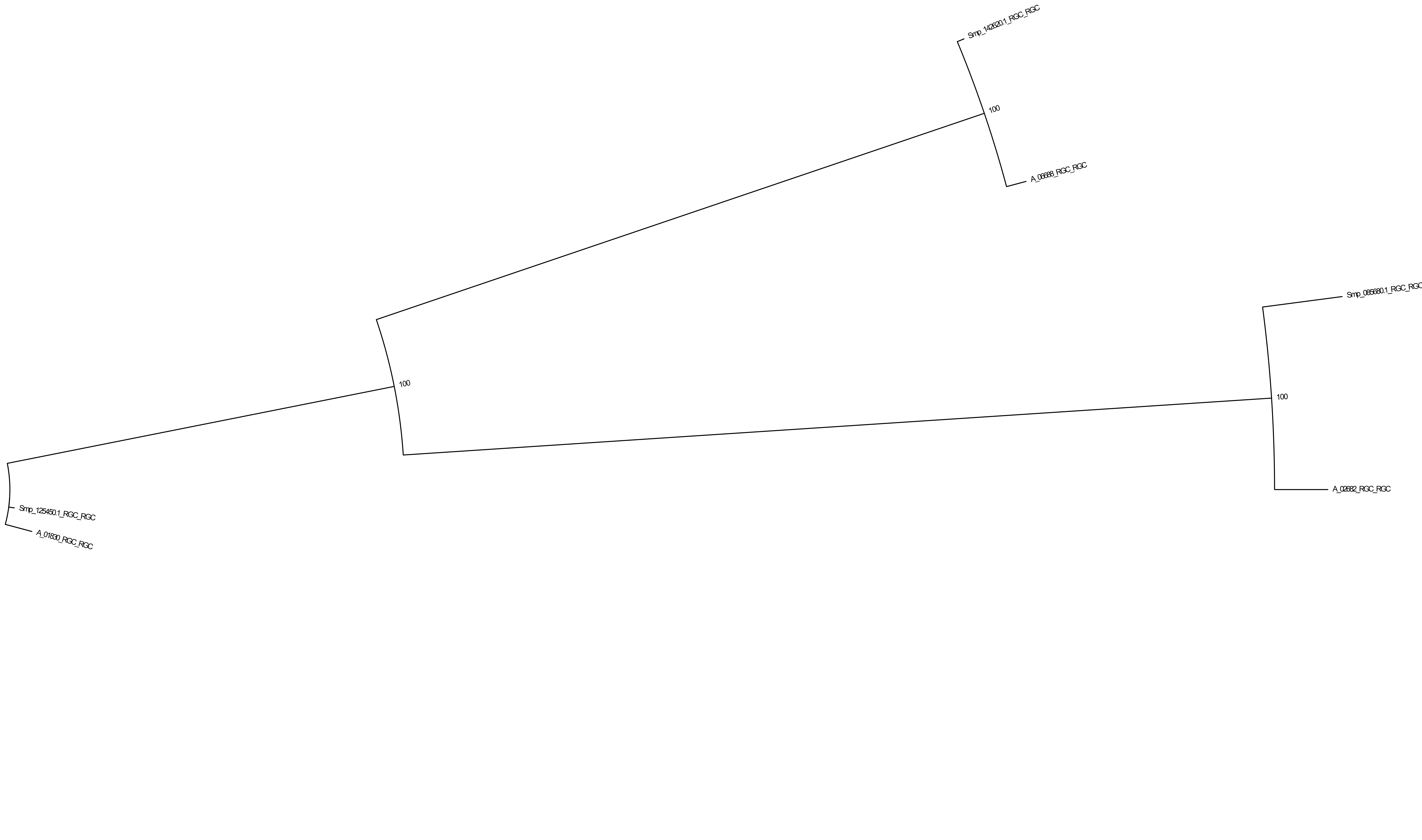

Supplementary Figure 10. RIO kinase family

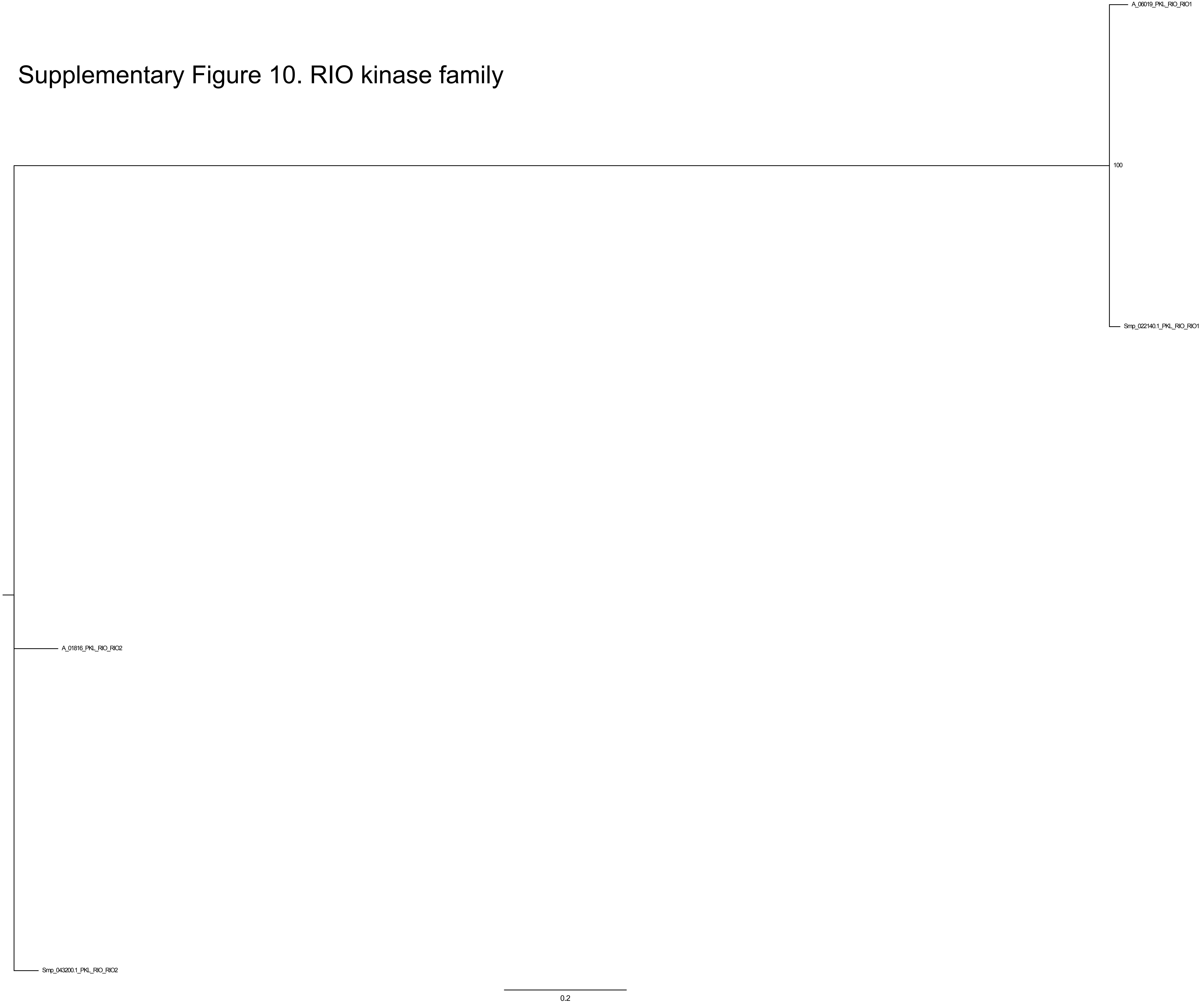

Supplementary Figure 11. ABC kinase family

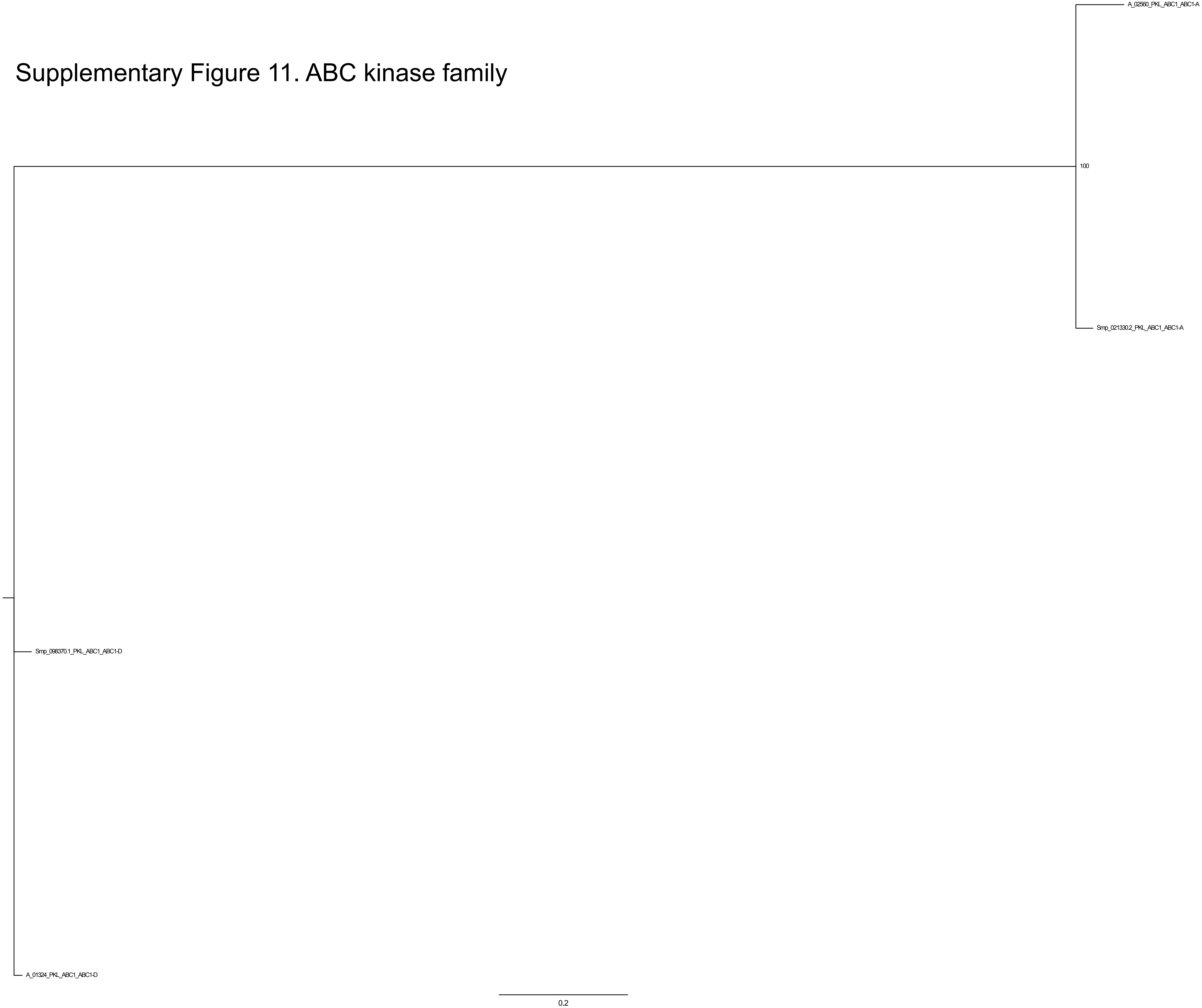

Supplement: Supplementary Figures 1-11 [file srep17759-s1.pdf]
